# Case Report: ^18^F-MK6240 Tau Positron Emission Tomography Pattern Resembling Chronic Traumatic Encephalopathy in a Retired Australian Rules Football Player

**DOI:** 10.3389/fneur.2020.598980

**Published:** 2020-12-22

**Authors:** Natasha Krishnadas, Vincent Doré, Fiona Lamb, Colin Groot, Paul McCrory, Rodney Guzman, Rachel Mulligan, Kun Huang, Meaghan O'Donnell, Jennie Ponsford, Malcolm Hopwood, Victor L. Villemagne, Christopher C. Rowe

**Affiliations:** ^1^Florey Department of Neuroscience and Mental Health, The University of Melbourne, Parkville, VIC, Australia; ^2^Department of Molecular Imaging & Therapy, Austin Health, Heidelberg, VIC, Australia; ^3^The Australian e-Health Research Centre, CSIRO Health & Biosecurity, Parkville, VIC, Australia; ^4^Department of Neurology, Alzheimer Center Amsterdam, Amsterdam Neuroscience, Vrije Universiteit Amsterdam, Amsterdam, Netherlands; ^5^The Florey Institute of Neuroscience and Mental Health, Parkville, VIC, Australia; ^6^Phoenix Australia, Parkville, VIC, Australia; ^7^The Department of Psychiatry, The University of Melbourne, Parkville, VIC, Australia; ^8^Monash-Epworth Rehabilitation Centre, Turner Institute for Brain and Mental Health, Monash University, Clayton, VIC, Australia

**Keywords:** Positron Emission Tomography (PET), Alzheimer's Disease (AD), tau, case report, Chronic Traumatic Encephalopathy (CTE)

## Abstract

**Introduction:** It remains unclear if tau imaging may assist diagnosis of chronic traumatic encephalopathy (CTE). Flortaucipir PET has shown superior frontal with medial temporal tau binding consistent with the provisional neuropathological criteria for mid-stage CTE in group-level analyses of retired symptomatic NFL players and in one individual with pathologically confirmed CTE. ^18^F-MK6240 is a new PET ligand that has high affinity for tau. We present the case of a 63-year-old cognitively impaired, former Australian rules football player with distinct superior frontal and medial temporal ^18^F-MK6240 binding and show it to be significantly different to the pattern seen in prodromal Alzheimer's disease (AD).

**Findings:** The participant was recruited for a study of amyloid-β and tau several decades after traumatic brain injury. He had multiple concussions during his football career but no cognitive complaints at retirement. A thalamic stroke in his mid 50s left stable mild cognitive deficits but family members reported further short-term memory, behavioral, and personality decline preceding the study. Imaging showed extensive small vessel disease on MRI, a moderate burden of amyloid-β plaques, and ^18^F-MK6240 binding in bilateral superior frontal and medial temporal cortices. Voxel-wise analysis demonstrated that the frontally predominant pattern of the participant was significantly different to the posterior temporo-parietal predominant pattern of prodromal AD.

**Conclusion:** Although lacking neuropathological examination to distinguish CTE from a variant of AD, the clear demonstration of a CTE-like tau pattern in a single at-risk individual suggests further research on the potential of ^18^F-MK6240 PET for identifying CTE is warranted.

## Introduction

Chronic traumatic encephalopathy (CTE) is a neurodegenerative tauopathy associated with repetitive concussive and sub-concussive head impacts, particularly at young ages (1).

CTE is currently a neuropathological diagnosis. The first consensus criteria require the presence of phosphorylated tau aggregates in neurons, astrocytes, and cell processes around small vessels in an irregular pattern at the depths of cortical sulci (2). CTE neuropathological features have been demonstrated in four retired professional Australian sports athletes (3–5). Three were former rugby players (3, 4), one of whom also had pTDP-43 and amyloid-β (Thal 1) co-pathology (4). The fourth, an Australian rules football player, was found to have features of stage III CTE with concurrent Alzheimer's disease (AD) neuropathologic change and severe small vessel disease (5). The authors highlighted the complexities of distinguishing tau pathology in cases of CTE concurrent with AD (5). In both CTE and AD, tau deposits in the form of neurofibrillary tangles (NFT) have been shown to comprise mixed 3-repeat/4-repeat isoforms (6), suggesting that the pattern of tau distribution rather than the presence of tau may be more useful in discriminating these two conditions. Proposed neuropathological stages of CTE describe initial cortical tau deposition in superior and dorsolateral frontal cortices (stage I), later involving frontal (more extensively), temporal, and inferior parietal cortices (stage II-III), followed by widespread cortical deposition (stage IV) (7). In contrast, tau deposition is predominantly posterior in AD, involving medial temporal and temporo-parietal cortices (8, 9). There remains debate as to whether the preliminary CTE neuropathological criteria are specific for prior head impacts or whether these findings can also occur in individuals without a history of head impacts (10).

There are no consensus criteria for the clinical characterization of CTE, though there have been many proposed (11–13). Some proposed clinical features, such as anger and depressive symptoms, are non-specific and commonly identified in community-dwelling individuals without a history of head injury (14). Additionally, clinical presentation alone may not be able to distinguish CTE from other conditions such as AD, particularly when these conditions are coexistent (15). With these caveats, traumatic encephalopathy syndrome (TES) is one proposed classification to characterize research participants based on cognitive, behavioral, mood, and motor symptoms or signs, as well as their functional state (13). The TES criteria are yet to be validated, although they present a provisional method of screening participants for relevant symptoms and also have a provision for the future inclusion of biomarkers.

Tau positron emission tomography (PET) is a potential *in vivo* biomarker of CTE. The early tau PET case reports of sports athletes used the tracer ^18^F-FDDNP, which is non-specific for tau, as it also binds amyloid-β plaques. Recently, a study using the tau tracer ^18^F-AV1451 (Flortaucipir) identified higher tracer retention in bilateral superior frontal, bilateral medial temporal, and left parietal regions in retired National Football League (NFL) players relative to controls, in group-level analyses (16). This frontotemporal pattern has also been reported in another group-level analysis of retired NFL players (17), but neither study had neuropathological confirmation of CTE. In both studies, the pattern was not a consistent feature at an individual level and the quantitative results overlapped with the control range. Recently, there has been a case report of a retired NFL player with neuropathological confirmation of CTE and superior frontal ^18^F-AV1451 binding; however, the correlation between the antemortem PET signal and quantitative tau burden on immunohistochemistry was limited (18). Ligands with higher affinity and selectivity for tau aggregates seen in CTE are required.

We present a case of a retired professional Australian rules football player with a clear fronto-temporal predominant pattern of tau deposition using a recently developed tau tracer ^18^F-MK6240. This 3R4R tau tracer has high target to background binding that may provide better sensitivity for detection of tau. To determine regions of significant difference in tau tracer retention in the case participant relative to healthy controls, a voxel-wise analysis was conducted. To determine whether this pattern was distinct from the spatiotemporal pattern seen in those on the AD continuum, voxel-wise analysis was also conducted between the case participant and participants with amyloid-β PET-assisted diagnoses of MCI due to AD (prodromal AD) and mild AD dementia. The case is also presented to underscore the need for ongoing research into biomarkers that are validated against postmortem diagnosis, given the complexity of clinically differentiating CTE from other neurodegenerative conditions, particularly in the presence of comorbidity and co-pathology.

### Case Description

The case participant is a 63-year-old male former Australian rules footballer, who played from the age of eight to retirement in his early 30s, including more than 200 professional games. He suffered several concussion injuries during his career (exact number unknown) including a period when he suffered three concussions in 4 weeks. He did not report any significant or ongoing cognitive or behavioral symptoms at the time of sustaining the concussion or at retirement. Following retirement from football, the participant worked in a coaching role. There was no history of exposure to other head impacts, no alcohol or drug abuse, and no family history of Alzheimer's disease (AD) or other dementia.

In his mid-50s, the participant had a stroke, which presented with features including non-fluent dysphasia and facial droop. An MR brain scan noted a 10-mm left thalamic infarct. Resulting short-term memory impairment necessitated a change to a less demanding occupation and he remains employed as a factory worker. His family reported gradual deterioration in his short-term memory post-stroke and recent behavioral change. His presentation was complicated by severe deafness and reluctance to use a hearing aid. Neurological examination was normal other than a loss of sense of smell.

## Materials and Methods

### Participants

The participant was recruited as part of a study on the impact of traumatic brain injury on the risk of later neurodegenerative disease. For comparison, 99 cognitively unimpaired amyloid-β PET-negative/tau PET-negative (A–/T–) healthy controls (HC) and 37 amyloid-β PET-positive/tau PET-positive (A+/T+) MCI and AD participants were utilized from the Australian Imaging Biomarkers and Lifestyle (AIBL) study (19). The study was approved by the institutional review board of Austin Health, Melbourne, Australia. Additional written informed consent was obtained from the participant to publish this report.

### Neuropsychological Assessment

All participants completed a subset of the AIBL neuropsychology battery (19). This comprised the Wechsler Test of Adult Reading; Mini-Mental State Examination (MMSE); Rey Complex Figure Test (RCFT); Delis–Kaplan Executive Function System—Verbal Fluency; Digit Span and Digit Symbol-Coding subsets of the Wechsler Adult Intelligence Scale—Third Edition. The case participant was administered the Rey Auditory Verbal Learning Test (RAVLT), while the comparison participants were administered the California Verbal Learning Test—Second Edition (CVLT-II) as a measure of verbal memory. The case participant also completed the Trail Making Task Part B.

### Clinical Assessment

All participants were administered the Clinical Dementia Rating (CDR) scale, which is validated as a measure of dementia severity, where 0 = absent, 0.5 = questionable, 1 = mild, 2 = moderate, and 3 = severe (20). All participants were administered the Hospital Anxiety and Depression Scale (HAD scale) to screen for depression and anxiety, where 0–7 was considered a low score (none-mild symptoms), 8–10 = moderate score, and 11–21 = high score (21).

The case participant's history of concussion was confirmed by witnesses and media reports. A semistructured interview was conducted to assess for TES symptoms, with questions adapted from the Montenigro et al. (13) publication (13). The case participant was asked to respond yes/ no, based on whether or not he endorsed any of the following (occurring subsequent to the time of his last most significant head impact): subjective memory loss; having a “short fuse;” feeling “out-of-control;” physical violence or verbal abuse; impulsivity; loss of interest in usual activities or loss of motivation; less likely to make plans or goals toward achieving a task; paranoia; or headaches (occurring more than once per month for a minimum of 6 months). If endorsed, a follow-up question was asked regarding onset and self-reported trajectory of the symptom, and the participant was permitted to elaborate on the response. His next-of-kin was interviewed separately, as a method of independently corroborating these responses.

The case participant was also administered an abbreviated Mini International Neuropsychiatric Interview version 7.02 (M.I.N.I.-7) for DSM-5, a structured diagnostic interview to screen for psychiatric disorders. The modules administered were as follows: A major depressive episodes and disorders; D panic disorder; E agoraphobia; F social anxiety disorder; H post-traumatic stress disorder; I alcohol use disorder; J substance use disorder; and N generalized anxiety disorder (22).

### Image Acquisition

Tau PET imaging involved IV administration of 185 MBq (±10%) ^18^F-MK6240 with a 20-min acquisition commencing 90-min post-injection (mpi). Amyloid-β PET imaging involved IV administration of 200 MBq (±10%) ^18^F-NAV4694 with a 20-min acquisition commencing 50 mpi. The case participant had PET scans acquired on a Siemens Biograph mCT, while AIBL comparison participants had scans on either this camera or a Philips TF64 PET/CT. Low-dose CT was used for attenuation correction. The case participant underwent a brain 3T MRI (sequences: T1-weighted and FLAIR) on a Philips Ingenia scanner. All other participants had 3T MRI on a Siemens Skyra or Prisma.

### Image Analysis

Amyloid-β and tau PET scans were coregistered and aligned with the individual's MRI T1-weighted scan and spatially normalized into MNI space using SPM8. The standard Centiloid method was applied for amyloid-β PET quantitation. A Centiloid value of >20 defined a positive scan.

For tau studies, a cortical gray matter inclusion mask plus a meninges exclusion mask were applied. Tau tracer SUVR was calculated using a large temporal cortical region (23) and cerebellar cortex as the reference. SUVR > 1.19 (reflecting the 95%ile of the amyloid-β negative HC) was used to discriminate between high and low tau burden. Tau SUVR was estimated in the dorsolateral prefrontal cortex (DLPFC) and three composite regions of interest (ROI): mesial temporal (comprising entorhinal cortex, hippocampus, parahippocampus and amygdala), temporo-parietal (comprising inferior temporal, fusiform, supramarginal and angular gyri, posterior cingulate/precuneus, superior and inferior parietal, and lateral occipital) (24) and frontal (comprising dorsolateral prefrontal, ventrolateral prefrontal, orbitofrontal, gyrus rectus, and anterior cingulate). Z-scores were used to compare the participant's SUVR and group mean (+SD) SUVR in each ROI.

In MNI space, the case participant's tau image was subtracted from each comparison participant's tau image. These subtracted images represent voxel-wise relative differences between ^18^F-MK6240 SUVR in the case and each of the comparison participants. By performing a one-sample *t*-test using the subtracted images of each of the comparison groups (HC, MCI, and AD) in SPM8, we could determine how much the case participant deviated from the group in ^18^F-MK6240 SUVR at a voxel-wise level. The threshold for significance was *p* < 0.001 uncorrected.

### Descriptive Statistics

Descriptive statistics are provided for all participants' demographic characteristics, Centiloid values, MMSE, CDR, and HAD scale scores (Table 1). All neuropsychological measures are presented as *z*-scores. Raw scores were converted to standardized *z*-scores using published normative datasets.

**Table 1:** Demographics and neuropsychological profile (participant and comparison groups).

	**Participant *n* = 1**	**A–/T– HC*n* = 99**	**A+/T+ MCI *n* = 16**	**A+/T+ AD*n* = 21**
**Age**				
Median (IQR)^a^	63	72.6 (70.4–75.4)	72.8 (69.0–76.4)	73.7 (66.0–77.6)
**Sex**				
Male, *n* (%)	Male	47 (47.5%)	11 (68.8%)	11 (52.4%)
**MMSE**				
Median (IQR)	29	29 (28–30)	26 (23–28)	23 (17–25)
**CDR**				
Mean (SD)	0.5	0.04 (0.13)	0.5 (0)	0.88 (0.44)
**Years of education**				
Median (IQR)	15	15 (12–16)	11 (10–15)	11 (10–15)
**Centiloid value**				
Median (IQR)	51	−0.6 (−3.5–3.6)	128 (100–148)	124 (96–141)
**HADS-D^b^**				
Median (IQR)	7.0	1.0 (1.0–2.0)	2.0 (1.0–3.3)	2.5 (1.0–5.8)
**HADS-A^c^**				
Median (IQR)	6.0	3.0 (2.0–4.3)	5.0 (4.0–7.0)	4.0 (2.0–6.0)
**Premorbid IQ**				
Median (IQR)	110	114 (109–117)	109 (102–111)	104 (101–112)
**Neuropsychological measures (*****z*****-scores)**				
Word list recall^d^	−2.27	1.13 (0.81)	−1.75 (0.82)	−2.18 (0.77)
RCFT copy score	−0.54	-0.98 (1.09)	−2.76 (2.83)	−4.63 (6.52)
RCFT (3-min delayed recall)	−2.31	1.14 (1.45)	−1.70 (0.67)	−2.22 (1.04)
RCFT (30-min delayed recall)	−3.85	1.21 (1.56)	−1.79 (0.69)	−2.32 (1.19)
COWAT	−0.66	0.97 (1.09)	-0.19 (1.19)	−0.20 (1.10)
Trail Making Task B	−0.33	–	–	–
Digit span	0.33	0.64 (1.01)	0.19 (1.09)	−0.44 (0.90)
Digit symbol coding	0.66	0.92 (0.82)	−0.33 (0.79)	−0.96 (0.99)

*A–/T–, amyloid-β negative, tau negative; A+/T+, amyloid-β positive, tau positive; MMSE, Mini-Mental State Examination; CDR, Clinical Dementia Rating scale; HADS-D, Depression subscale of the Hospital Anxiety and Depression Scale; HADS-A, Anxiety subscale of the Hospital Anxiety and Depression Scale; RCFT, Rey Complex Figure Test; COWAT, Controlled Oral Word Association Test (component of the Delis-Kaplan Executive Function System verbal fluency test)*.

*Mean (SD) for all neuropsychological z-scores*.

a *The case participant was 63 years of age at the time of neuropsychological assessment, amyloid, and tau imaging*.

b *HADS-D: raw score; 0–7 = low score (none–mild symptoms); 8–10 = moderate; 11–21 = high (21)*.

c *HADS-A: raw score; 0–7 = low score (none–mild symptoms); 8–10 = moderate; 11–21 = high (21)*.

d *Participant was administered the Rey Auditory Verbal Learning Test (RAVLT), while comparison groups were administered the California Verbal Learning Test (CVLT)*.

## Results

Comparison groups were older than the participant, with MMSE and CDR scores consistent with their diagnostic categories (Table 1).

### Neuropsychological Findings

The participant had impairments in new learning and memory in both visual and verbal domains, as evidenced by performances more than two standard deviations (SD) below the normative level on the Rey Complex Figure Test (RCFT) and word list recall (RAVLT) (Table 1). His attention, concentration, and executive function skills scored within one SD of the norm, suggesting they were relatively preserved.

### Clinical Interview

The participant endorsed subjective memory loss. He had 1–2 years of behavioral and personality change, characterized by irritability and anger outbursts. He endorsed having a “short-fuse” and feeling at times “out of control,” but there were no episodes of physical violence. He reported loss of interest in his usual activities, amotivation, and paranoia. Corroborative history revealed he had become increasingly impulsive and withdrawn, with a propensity to fixate on individuals and ideas. There was no history of headaches. The participant was still employed, continued to drive, and was independent in daily activities. The participant had a similar level of functional impairment as the amyloid and tau PET positive (A+/T+) MCI comparison group (CDR 0.5) and had a CDR sum of boxes of 0.5. He did not endorse feelings of sadness/depression/hopelessness or anxiety and did not meet psychiatric diagnostic criteria on any of the M.I.N.I.-7 modules. Scores on both the anxiety and depression scales of the HAD scale were in the mild range, and no suicidality was identified on the M.I.N.I.-7.

### Tau PET Voxel-Wise Analysis

**Figure 1:**
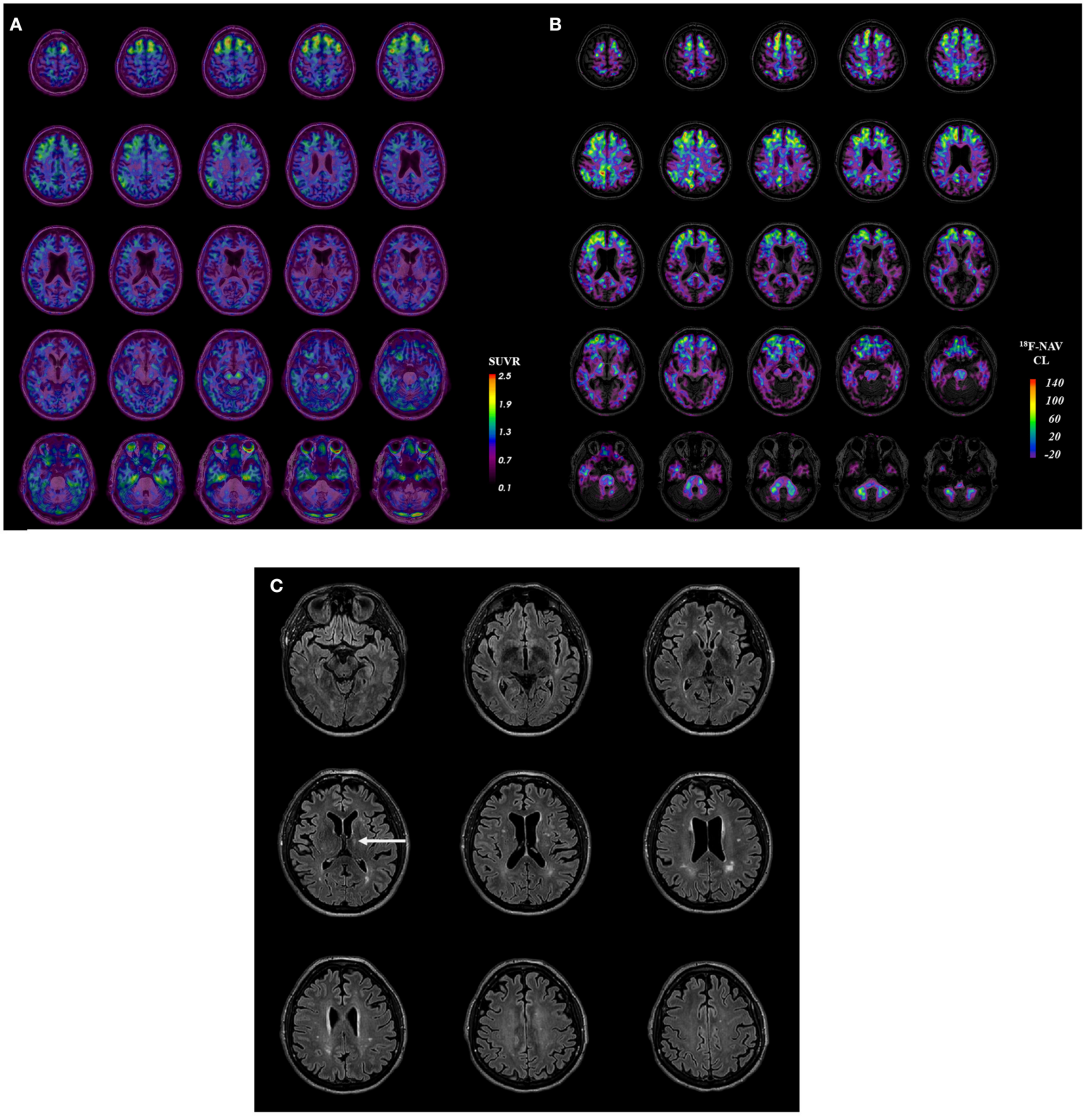
Case participant's. **(A)** Tau PET image. **(B)** Amyloid PET image. **(C)** Axial FLAIR image. Participant **(A)** ^18^F-MK6240 tau PET image in SUVR (standardized uptake value ratio; using the cerebellar cortex as the reference region) coregistered onto T1 MRI. **(B)** Participant's ^18^F-NAV4694 amyloid PET image in the Centiloid scale. **(C)** Participant's MRI (representative axial FLAIR sequences) with the arrow indicating his prior left thalamic infarct.

**Figure 2:**
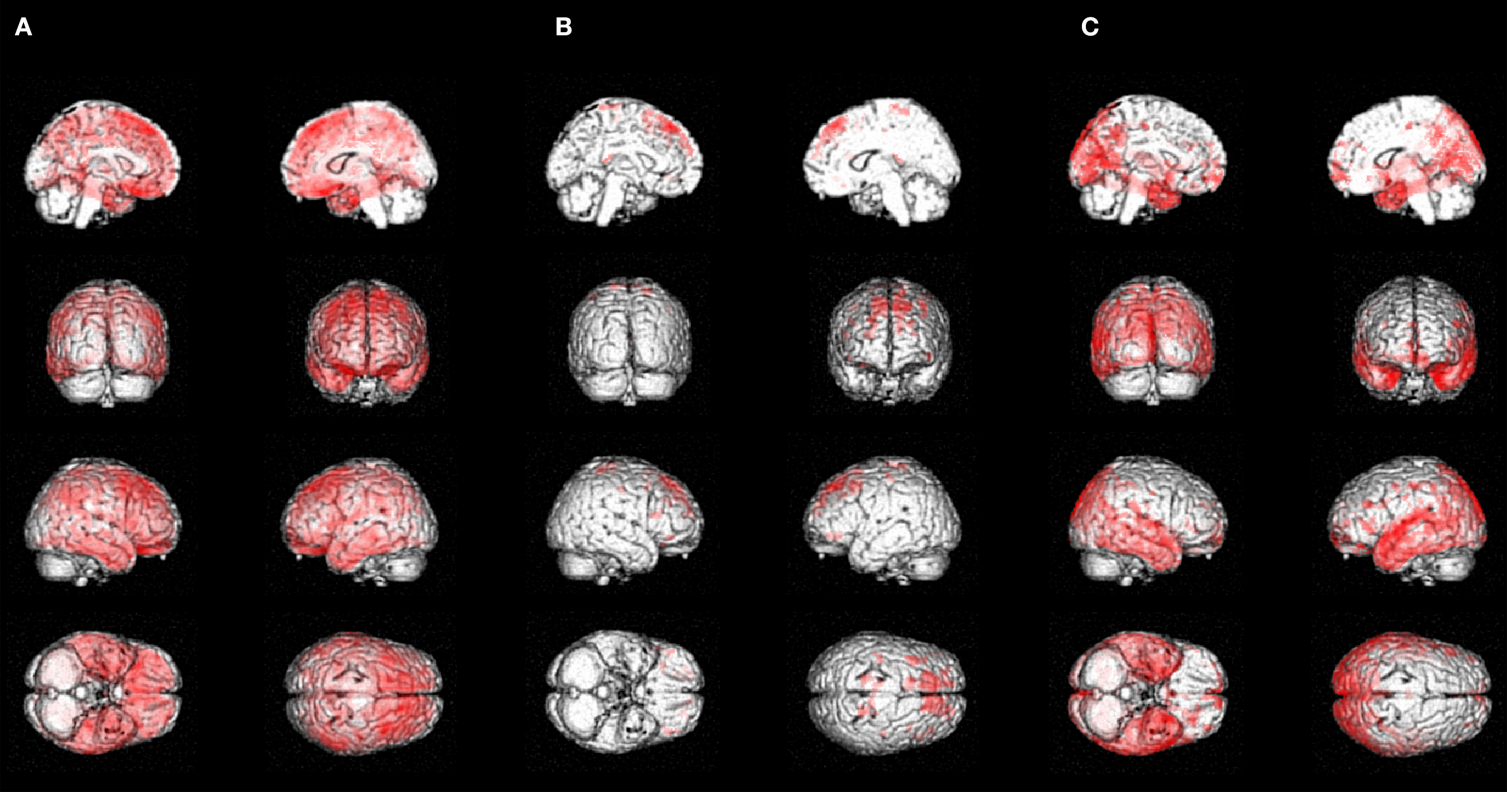
Voxel-wise analysis: **(A)** Case compared to controls. **(B)** Case compared to prodromal AD **(C)** Prodromal AD compared to case. Significant voxels (*p* < 0.001, uncorrected) from a one-sample *t*-test for the: **(A)** negative contrast on the SUVR subtracted image [HC–participant] (regions of higher tau retention in the participant relative to A–/T– HC). **(B)** negative contrast on the SUVR subtracted image [MCI–participant] (regions of higher tau retention in the participant relative to the A+/T+ MCI comparison group). **(C)** positive contrast on the SUVR subtracted image [MCI–participant] (regions of higher tau retention in the A+/T+ MCI comparison group relative to the participant).

The participant had high retention in bilateral superior frontal gyri particularly in the dorsolateral prefrontal cortex (DLPFC) and medial temporal cortex. Mild focal uptake was also present in the right supramarginal, right orbitofrontal, and bilateral lateral temporal gyri (Figure 1A), significantly higher than the A–/T– HC (Figure 2A). The participant had higher retention in bilateral superior frontal regions compared to the A+/T+ MCI group (Figure 2B), while at the same threshold, the A+/T+ MCI group had higher retention in medial temporal and temporo-parietal regions (Figure 2C) (Supplementary Figure 1). The A+/T+ AD group had higher retention in all cortical regions compared to the participant.

### Tau PET Region-of-Interest Analysis

The participant had higher SUVR in all ROI compared to A–/T– HC (>4 SD higher in the DLPFC) and higher SUVR in the DLPFC compared to the A+/T+ MCI group (0.70 SD), while the participant had lower SUVR in mesial temporal and temporo-parietal composite ROI than the A+/T+ MCI group (−1.96, −1.35 SD) (Table 2).

**Table 2:** Tau region-of-interest analysis.

**ROI**	**Case Participant**	**A–/T– HC**	**A+/T+ MCI**	**A+/T+ AD**
DLPFC SUVRcbcx	1.56	0.91 (0.16)^*^	1.23 (0.47)	2.17 (1.10)
Frontal composite SUVRcbcx	1.08	0.84 (0.12)^*^	1.13 (0.30)	1.73 (0.72)
Me composite SUVRcbcx	0.97	0.85 (0.13)	1.99 (0.52)^**^	2.34 (0.71)
Te composite SUVRcbcx	1.16	1.00 (0.13)	1.85 (0.51)	2.97 (1.27)

*DLPFC, dorsolateral prefrontal cortex; Me, mesial temporal; Te, temporo-parietal; SUVRcbcx, standardized uptake value ratio using the cerebellar cortex as the reference region; A-/T-, amyloid-β negative, tau negative; A+/T+, amyloid-β positive, tau positive*.

Participant's tau SUVR compared to the mean (+/–SD) SUVR for each group in each ROI. Z-score comparison between SUVR for the case participant and each comparison group, where

* = Z-score >1.96 and

** *= Z-score <-1.96*.

### Amyloid PET and MRI

The participant had a moderate burden of amyloid-β plaques (Centiloid 51) (Figure 1B). MRI showed a moderate burden of white matter hyperintensities and evidence of the prior left thalamic infarct (Figure 1C). There were no abnormalities of the septum pellucidum.

## Discussion

We present this case of a retired professional Australian rules football player with documented multiple concussions and cognitive and behavioral changes several decades later to highlight an unusual fronto-temporal predominant pattern of tau deposition on PET imaging that may suggest CTE. This pattern was clearly shown in this individual using a new tau tracer ^18^F-MK6240. This pattern of tau tracer distribution has been reported in group-level analyses of symptomatic retired NFL players imaged with Flortaucipir (16, 17) and a case report of a retired NFL player with CTE confirmed by neuropathological examination (18). However, quantitative tau results were within the range of control participants in the case–control studies, and limited antemortem–postmortem correlation was observed in the case study.

To determine if this pattern was distinctive, we compared it to the tau pattern in A+/T+ MCI (prodromal AD) and mild dementia due to AD participants. The progression of tau deposition in AD is topographically stereotyped, consistent with Braak staging (8) and Delacourte stages (9), with frontal lobe involvement seen in more advanced stages (typically Braak V–VI; Delacourte 7–10). Compared to A+/T+ MCI participants, who had a similar degree of functional impairment, the participant had higher frontal retention and significantly less retention in areas that usually precede frontal tau, i.e., mesial, anterior, inferior, and mid-temporal (Braak I–IV; Delacourte 1–6). The participant's regional tau deposition was inconsistent with the typical distribution of tau in AD and more consistent with CTE stages (7). The A+/T+ AD group, had more advanced stages of tau deposition including frontal regions (consistent with Braak stages IV–VI; Delacourte stages 7–10). However, overall, the participant's frontally predominant tau pattern differs from the posterior temporo-parietal pattern typical of AD.

The moderate burden of amyloid-β plaques in this case does not differentiate between CTE and AD. Neuritic plaque co-pathology has been observed in 36% of CTE cases, where the presence of amyloid-β plaques has been reported to be associated with a more severe stage of CTE neuropathology (25). Although both CTE and AD are characterized by 3R/4R tau, a distinct difference in fibril structure has been observed using cryo-electron microscopy (26). It is unclear if this difference is sufficient to impact the binding affinity of these ligands to tau aggregates of CTE, as these compounds were screened based on their binding affinity to NFT in AD brain homogenates. *In vitro* studies are lacking. A single autoradiography study reports no detectable ^18^F-MK6240 binding in CTE; however, it is limited by the small number of CTE cases (*n* = 5) (27). Validation in a larger sample of neuropathologically confirmed CTE cases is needed. Therefore, while the tau PET pattern in this case is consistent with the described pattern of CTE, in the absence of neuropathological confirmation, we cannot exclude the possibility that this case represents an atypical, frontally predominant AD variant.

This study is limited in that the case participant was younger than the comparison group participants (on average, 10 years younger than the A+/T+ AD group). As the case participant was 63 years of age at the time of tau imaging, there was an insufficient number of A+/T+ MCI and AD AIBL participants <65 to permit age matching (*n* = 2).

The clinical presentation in this case is complex. The participant had primarily amnestic cognitive impairment without objective frontal lobe dysfunction on neuropsychological testing, which could be consistent with his prior thalamic stroke, prodromal AD, or CTE. The relatively recent onset of behavioral symptoms suggests a progressive disease process. The finding of amyloid plaques and tau on PET imaging at the age of 63 years is strong evidence that a neurodegenerative disease, either CTE or AD, is present in addition to the vascular disease seen on MRI. In the relatively nascent field of CTE research, this case highlights the challenges of reconciling clinical symptoms, exposures to head impacts, neuropsychological measures, and biomarkers in a condition where comorbidity and co-pathology is anticipated to be more common than single neuropathology.

## Conclusion

The aim of the case report is to present a clear individual example of the superior frontal plus medial temporal predominant tau PET pattern in a retired professional football player, which may be indicative of CTE. The pattern of tau deposition may be critical in differentiating CTE from AD, as both have tau aggregates comprised of 3R/4R isoforms and amyloid-β plaques may be present in both. However, tau PET biomarkers and the specificity of a frontal–medial temporal pattern for CTE require further postmortem validation. In this case, while this tau PET pattern was significantly different to CDR-matched AD patients, suggesting it may represent CTE, in the absence of neuropathological confirmation, a frontally predominant AD variant cannot be excluded.

## Ethics Statement

The studies involving human participants were reviewed and approved by the institutional review board of Austin Health, Melbourne, Australia. The patients/participants provided their written informed consent to participate in this study. Written informed consent was obtained from the individual(s) for the publication of any potentially identifiable images or data included in this article.

## Author Contributions

VV and CR contributed to the conception and design of the study and edited the manuscript. NK contributed to data acquisition, image analysis, and wrote the manuscript. VD and CG contributed to image analysis and interpretation. FL, RG, RM, and KH significantly contributed to data acquisition. PM, MO'D, JP, and MH contributed to the conception and design of the overarching TBI study from which the case participant was recruited. PM conducted a detailed physical examination and obtained consent from the case participant for publication. All authors contributed to manuscript revision and read and approved the submitted version.

## Conflict of Interest

PM is a co-investigator on competitive grants relating to mild TBI funded by several governmental and other organizations. He is funded under a Fellowship awarded by the National Health & Medical Research Council of Australia and is employed at the Florey Institute of Neuroscience and Mental Health. He has a clinical consulting practice in neurology, including medico-legal work. He has been reimbursed by the government, professional scientific bodies, and commercial organizations for discussing or presenting research relating to MTBI and sport-related concussion at meetings, scientific conferences, and symposiums. He does not hold any individual shares in or receive monies from any company related to concussion or brain injury assessment or technology. He acknowledges unrestricted philanthropic support from CogState Inc. (2001-16). He is the chair of the scientific committees of the International Concussion and Head Injury Research Foundation in London and the Sports Surgery Clinic in Dublin. PM did not receive any form of financial support directly related to this manuscript. CR was the recipient of a research grant from Cerveau who supplied the MK6240 tau tracer precursor for research use. He has received research grants from the NHMRC and US Department of Defense to study biomarkers of Alzheimer's disease decades after traumatic brain injury. The remaining authors declare that the research was conducted in the absence of any commercial or financial relationships that could be construed as a potential conflict of interest. The reviewer GR declared a past co-authorship with several of the authors VD, CG, VV, CR to the handling Editor.

## Supplementary Material

Supplementary Figure 1 A+/T+ MCI Group Mean ^18^F-MK6240 tau PET image. A+/T+ MCI group mean ^18^F-MK6240 tau PET image coregistered onto a T1 MRI template.
