# Supplementary figures and images for: Case Report: 18F-MK6240 Tau Positron Emission Tomography Pattern Resembling Chronic Traumatic Encephalopathy in a Retired Australian Rules Football Player
Source: Front Neurol. 2020 Dec 22;11:598980. doi: 10.3389/fneur.2020.598980 (PMC7783156; doi:10.3389/fneur.2020.598980)

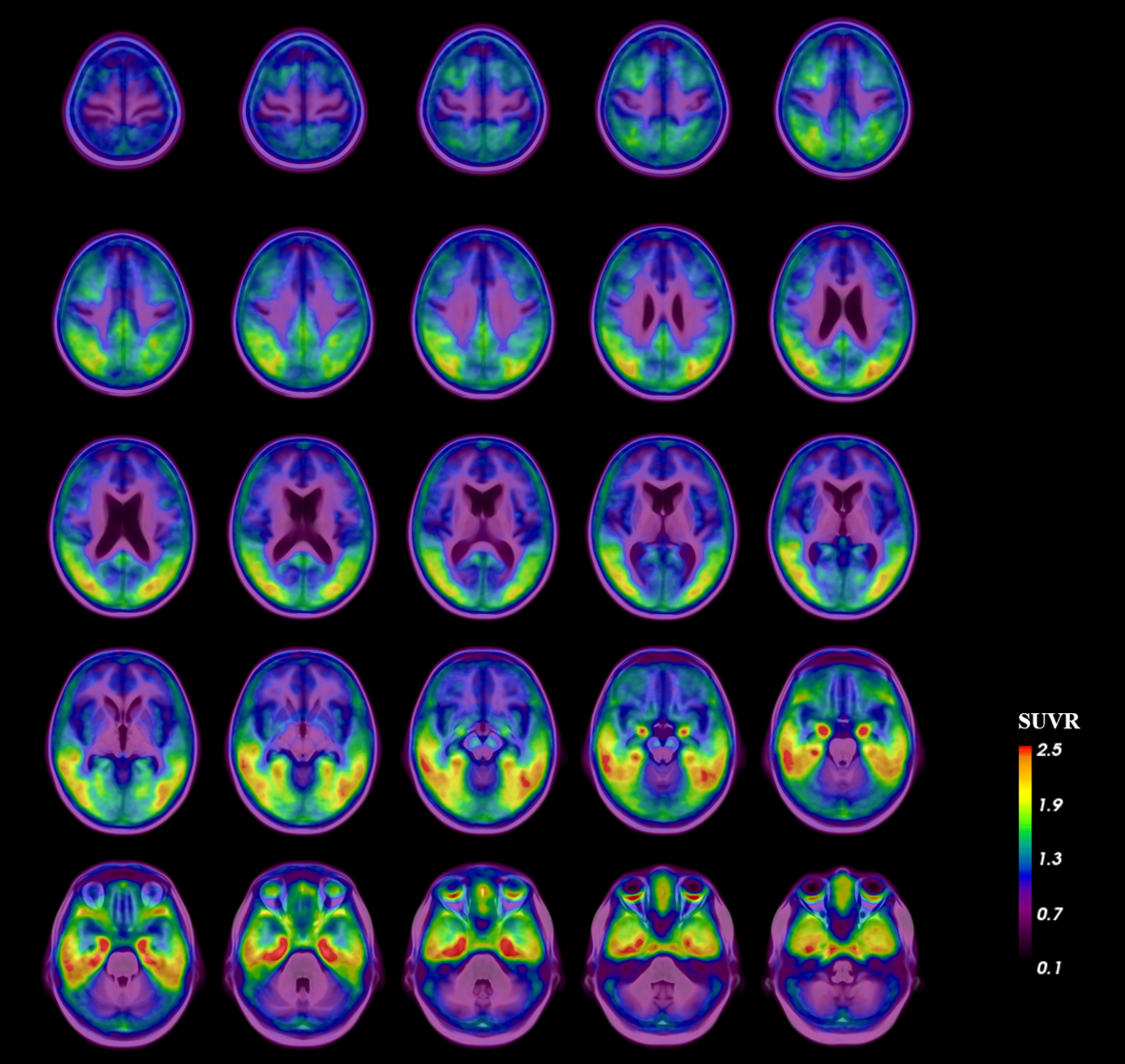

Supplement: Supplementary Figure 1 — A+/T+ MCI Group Mean 18F-MK6240 tau PET image. A+/T+ MCI group mean 18F-MK6240 tau PET image coregistered onto a T1 MRI template. [file Image_1.TIFF]
